# Genetically Encoded Libraries of Nonstandard Peptides

**DOI:** 10.1155/2012/713510

**Published:** 2012-10-14

**Authors:** Takashi Kawakami, Hiroshi Murakami

**Affiliations:** Department of Life Sciences, Graduate School of Arts and Sciences, The University of Tokyo, Meguro-ku, Tokyo 153-8902, Japan

## Abstract

The presence of a nonproteinogenic moiety in a nonstandard peptide often improves the biological properties of the peptide. Non-standard peptide libraries are therefore used to obtain valuable molecules for biological, therapeutic, and diagnostic applications. Highly diverse non-standard peptide libraries can be generated by chemically or enzymatically modifying standard peptide libraries synthesized by the ribosomal machinery, using posttranslational modifications. Alternatively, strategies for encoding non-proteinogenic amino acids into the genetic code have been developed for the direct ribosomal synthesis of non-standard peptide libraries. In the strategies for genetic code expansion, non-proteinogenic amino acids are assigned to the nonsense codons or 4-base codons in order to add these amino acids to the universal genetic code. In contrast, in the strategies for genetic code reprogramming, some proteinogenic amino acids are erased from the genetic code and non-proteinogenic amino acids are reassigned to the blank codons. Here, we discuss the generation of genetically encoded non-standard peptide libraries using these strategies and also review recent applications of these libraries to the selection of functional non-standard peptides.

## 1. Introduction

Nonstandard peptides, also known as unnatural peptides or peptidomimetics, are peptide-based small molecules containing a moiety that does not exist in standard (i.e., natural) peptides composed of only 20 proteinogenic amino acids. The nonproteinogenic moiety in nonstandard peptides, such as a nonproteinogenic side chain, a modified backbone, or a macrocyclized backbone, often contributes to improving the peptide's cell permeability, stability against peptidases, and conformational rigidity, thereby affording specific high affinity toward its target molecule [1–5]. Naturally occurring nonribosomal peptides (e.g., immunosuppressant cyclosporine A) are representative of nonstandard peptides, and given the success of nonribosomal peptides as therapeutics, the development of methods to construct highly diverse drug-like nonstandard peptide libraries is important for the discovery of novel drug candidates. Chemical synthesis can generate highly modified drug-like nonstandard peptide libraries, but the size of these libraries is relatively small (with a diversity of up to 10^6^ unique compounds). In contrast, by using genotype-phenotype linking technology, ribosomal synthesis can generate genetically encoded peptide libraries with extremely high diversity (up to 10^13^ compounds) [6–9]. However, ribosomally synthesized peptide libraries are typically composed of just the 20 proteinogenic amino acids. This had led to the development of various strategies to generate highly diverse nonstandard peptide libraries; these strategies integrate biology-based methods to construct highly diverse libraries and chemistry-based methods to construct highly modified drug-like libraries.

One approach to generate genetically encoded nonstandard peptide libraries involves the posttranslational modification of ribosomally synthesized peptide libraries. Examples include using chemical or enzymatic reactions for the posttranslational macrocyclization of standard peptide libraries. In the chemical cyclization approach, Heinis and coworkers used 1, 3, 5-tris(bromomethyl)benzene to react with three cysteine residues in peptide libraries displayed on phages, and the resulting bicyclic peptide libraries were used to obtain bicyclic peptide inhibitors against several enzymes [10–12]. In the enzymatic cyclization approach, Bosma et al. reported the bacterial display of peptide libraries cyclized with a thioether-bridge that was posttranslationally added using enzymes, and demonstrated thioether-bridged peptide selection against a model protein (streptavidin) [13]. Another example of the posttranslational conversion of standard peptide libraries to nonstandard ones is the modification of peptides with a moiety that selectively interacts with a target protein. Li and Roberts chemically modified a peptide library by reacting the sulfhydryl group of cysteine residues in the library with a bromoacetamide derivative of penicillin, and the penicillin-modified peptide library was subjected to mRNA display selection against penicillin binding protein 2a [14]. An excellent review discussing these posttranslational strategies has recently been published elsewhere [15].

**Figure 1:**
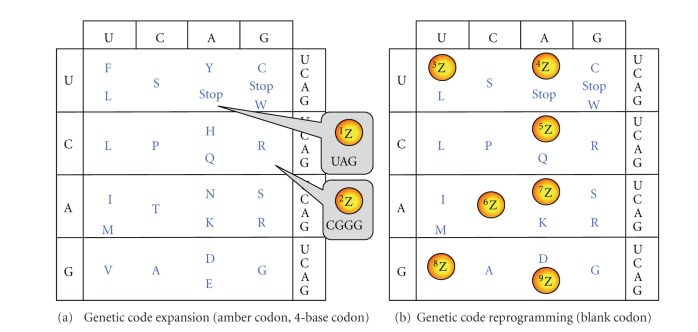
Generation of genetically encoded libraries of nonstandard peptides. (a) Genetic code expansion. Nonproteinogenic amino acids are assigned to the amber codon and four-base codons. (b) Reprogramming the genetic code. Nonproteinogenic amino acids are reassigned to blank codons generated by reconstructing a cell-free translation system with a reduced number of amino acids and protein factors. ^*n*^Z (*n* = 1–9) represents one of the nonproteinogenic amino acids.

Another approach to generate genetically encoded nonstandard peptide libraries involves the engineering of a ribosomal translation system, that is, cotranslational incorporation of nonproteinogenic amino acids into ribosomally synthesized polypeptides (Figure 1). This approach is further divided into two strategies: genetic code expansion and genetic code reprogramming. In this paper, we discuss both strategies and their application for the generation of genetically encoded nonstandard peptide libraries as well as the use of these libraries for the selection/evolution of functional nonstandard peptides.

## 2. Genetic Code Expansion

The genetic code expansion strategy (Figure 1(a)) has been used mainly for the study of proteins rather than short peptides because it allows the site-specific incorporation of a 21st amino acid in addition to the 20 proteinogenic amino acids. In 1989, the amber suppression method, the first example of a genetic code expansion strategy, was independently reported by Schultz's group and Chamberlin's group (Figure 1(a)) [16, 17]. In these reports, amber suppressor tRNA(CUA) carrying a nonproteinogenic amino acid was synthesized by chemoenzymatic acylation, which was developed by Hecht's group [18], and the resulting aminoacyl-tRNA (aa-tRNA) was added to an *in vitro* translation system (conventional crude lysate system). Accordingly, a polypeptide containing the nonproteinogenic amino acid was synthesized from an mRNA bearing a site-specifically mutated amber UAG codon to incorporate the nonproteinogenic amino acid. Later, Chamberlin and coworkers also reported that the other nonsense codons (opal UGA codon and ochre UAA codon) could be suppressed effectively by the corresponding nonproteinogenic aa-tRNA in rabbit reticulocyte lysate [19]. The extension of this nonsense suppression method to use a four-base codon was reported by Sisido's group. In this approach, a nonproteinogenic aa-tRNA bearing an ACCU four-base anticodon was used to incorporate a nonproteinogenic amino acid at an AGGU four-base codon [20]. They also expanded this approach to other four-base [21–23] and even five-base codons [24] and showed the simultaneous incorporation of multiple nonproteinogenic amino acids into proteins [25–28]. Later, the genetic code expansion strategy was further extended from *in vitro* to *in vivo*. In 1995, Dougherty, Lester, and coworkers reported *in vivo* nonsense suppression, where UAG-containing mRNA and suppressor tRNA, which was chemically acylated with a nonproteinogenic amino acid, were injected into a *Xenopus* oocyte to incorporate the nonproteinogenic amino acid into an ion channel expressed on the surface of the oocyte [29, 30]. Further progress came with the development of orthogonal tRNA(CUA) and aminoacyl-tRNA synthetase (aaRS) pairs, which produce an orthogonal tRNA(CUA) carrying a nonproteinogenic amino acid *in vivo* under multiple turnover conditions. This technology allows us to not only synthesize a protein carrying a nonproteinogenic amino acid at a specific position in various types of cells, but also obtain a protein containing a nonproteinogenic amino acid in a larger quantity than by *in vitro* genetic code expansion [31–34]. The use of various orthogonal tRNA/aaRS pairs has allowed for the synthesis of proteins carrying various artificial functional groups, such as a biochemical group (e.g., sulfate, acetate, or methylate) [35–38], fluorescent probe [39–43], photo-cross-linker [44–49], photo-caged group [50–56], and bioorthogonal reactive group [57–63], for the study of protein structure and function. Similarly to *in vitro* genetic code expansion, *in vivo* amber suppression using orthogonal tRNA/aaRS pairs was extended to ochre and opal codon suppression [64–66] and four-base codon suppression to incorporate two nonproteinogenic amino acids simultaneously into proteins *in vivo* [67, 68].

## 3. Selection of Functional Nonstandard ****Peptides Generated by Genetic Code ****Expansion

**Figure 2:**
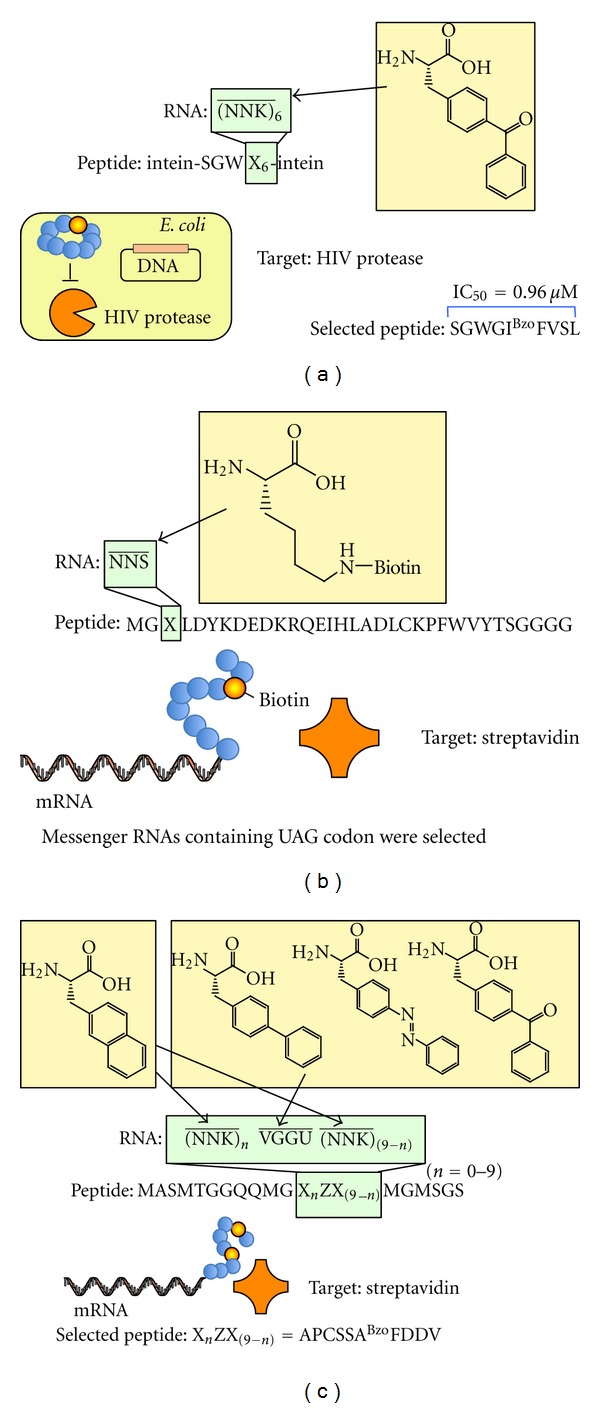
Selection of nonstandard peptides from libraries generated by genetic code expansion. (a) Selection of backbone-cyclized L-*p*-benzoylphenylalanine-containing peptide inhibitors. Orthogonal tRNA(CUA)/aaRS pairs were expressed in *E. coli* in order to assign L-*p*-benzoylphenylalanine to the amber codon. SICROPPS was used to cyclize the peptides. (b) Proof-of-concept study of mRNA display with a nonproteinogenic amino acid. Biocytin was assigned to the amber codon by adding biocytin-tRNA(CUA) to a rabbit reticulocyte lysate. The peptides were expressed in mRNA display format and pulled down using streptavidin beads. (c) Selection of streptavidin binding non-standard peptides. Four nonproteinogenic amino acids were assigned to the amber codon and four-base codons (UAG, L-2-naphthylalanine; AGGU, L-*p*-biphenylalanine; CGGU, L-*p*-benzoylphenylalanine; GGGU, L-*p*-phenylazophenylalanine). All corresponding nonproteinogenic aa-tRNAs were added to an *E. coli* cell-free translation system. The peptide libraries were expressed in mRNA display format and used for *in vitro* selection against streptavidin. X represents one of the proteinogenic or nonproteinogenic amino acids, and Z represents one of the nonproteinogenic amino acids.

The *in vivo* genetic code expansion strategy has recently been used to generate nonstandard peptide libraries. Using orthogonal aaRS/tRNA(CUA) pairs, Young et al. generated a backbone-cyclized peptide library containing a nonproteinogenic amino acid and performed in-cell selection of a nonstandard peptide inhibitor from the library (Figure 2(a)) [69]. The orthogonal aaRS/tRNA(CUA) pairs were expressed in *Escherichia coli* (*E. coli)* in order to assign L-*p*-benzoylphenylalanine to the amber codon [46]. Additionally, the split intein catalyzed ligation of proteins and peptides (SICROPPS) method, which was developed by Benkovic's group [70, 71], was used to cyclize the backbone of a ribosomally synthesized peptide library containing L-*p*-benzoylphenylalanine. The activity-based in-cell selection of human immunodeficiency virus (HIV) protease inhibitors from the cyclic peptide library was performed by linking the protease inhibitory activity of a cyclic peptide to cell viability of the host *E. coli*. The most abundant peptide obtained after two rounds of selection inhibited HIV protease activity at a low micromolar concentration (IC_50_ = 0.96 *μ*M). Interestingly, the L-*p*-benzoylphenylalanine residue of the peptide formed a covalent Schiff-base adduct with the *ε*-amino group of Lys14 of the HIV protease. 

Although this study using *in vivo* selection succeeded in selecting new functional nonstandard peptides, the size of the library was limited by the transformation efficiency of *E. coli*. In contrast, *in vitro* display methods, such as ribosome display [7] and mRNA display (or *in vitro* virus) [8, 9], can generate highly diverse libraries (up to 10^13^ compounds) because library construction does not involve cell transformation. Moreover, *in vitro* genetic code expansion has an advantage over *in vivo* expansion because membrane-impermeable, metabolically unstable or cytotoxic nonproteinogenic amino acids can be used as the building blocks for genetically encoded nonstandard peptides. The first proof of concept study of an *in vitro* display selection from a library containing a nonproteinogenic amino acid was reported by Li et al. (Figure 2(b)) [72, 73]. A nonstandard peptide library displayed on mRNA was prepared by using a lysate translation system containing amber suppressor tRNA(CUA) precharged with biocytin and a puromycin-modified mRNA library containing one NNS (S = C or G) codon. The authors demonstrated that mRNA containing the UAG codon (i.e., encoding a biocytin-containing peptide) was selectively recovered from the prepared library by pulldown using streptavidin beads. The same group combined a similar genetic code expansion strategy with a posttranslational chemical cyclization method to generate a nonstandard cyclic peptide library [74]. A peptide library containing *N*-methyl-L-phenylalanine assigned to the amber codon was displayed on mRNA and chemically converted to a cyclic peptide library by linking the *N*-terminal *α*-amino group to the *ε*-amino group of a lysine residue using disuccinimidyl glutarate. This library was used to select G*α*i1 binding peptides, and the best binding cyclic peptide had very high affinity against G*α*i1 (2.1 nM). Although this cyclic peptide also showed higher proteolytic stability than its linear counterpart, the *in vitro*-selected peptides in this study unfortunately did not contain the nonproteinogenic amino acid. Muranaka et al. also reported the selection of a streptavidin-binding peptide from nonstandard peptide libraries prepared by combining the amber and four-base codon suppression approaches (Figure 2(c)) [75]. Messenger RNA libraries composed of nine NNK (K = U or G) codons and one VGGU (V = C, A, or G) four-base codon were used to construct non-standard peptide libraries in the mRNA display format, and a streptavidin-binding non-standard peptide was successfully selected. The selected peptide contained only one nonproteinogenic amino acid (L-*p*-benzoylphenylalanine) residue (Figure 2(c)), even though multiple nonproteinogenic amino acids could have appeared in the sequence of the used libraries.

## 4. Limitation of Genetic Code Expansion for the Preparation of Nonstandard Peptide Libraries

In principle, highly modified non-standard peptides containing multiple nonproteinogenic amino acids can be produced by genetic code expansion, but this strategy actually imposes considerable restrictions on the synthesis of such peptides. In this strategy, the incorporation of nonproteinogenic amino acids competes with other normal events during translation. For example, an amber suppressor nonproteinogenic aa-tRNA(CUA) competes with endogenous release factor 1 (RF-1), which results in the production of truncated as well as full-length peptides from one mRNA template [16, 17]. Similarly, a 4-base suppressor nonproteinogenic aa-tRNA(ACCU) competes with endogenous Arg-tRNA^Arg^(CCU) for decoding the AGG(U) codon, which results in the production of a mixture of in-frame and out-of-frame products from one mRNA template [20]. This feature makes it difficult to simultaneously incorporate multiple nonproteinogenic amino acids into a peptide since the yield of a full-length (in-frame) peptide decreases exponentially as the number of nonproteinogenic amino acids increases. Moreover, in these genetic code expansion strategies, an mRNA containing the nonsense or 4-base codon does not encode one peptide but a mixture of multiple peptide products. This could be a more critical problem for peptide selection because this phenomenon would make it difficult to identify an active species from selected cDNA sequences. Recently, as one of the approaches used to decrease these competing translation events, Chin and coworkers evolved an orthogonal mutant ribosome that is thought to have a decreased functional interaction with RF-1 and demonstrated the increased incorporation efficiency of a nonproteinogenic amino acid on the amber codon [76]. However, since the orthogonal mutant ribosome was still recognized by RF-1 to some extent, a mixture of full-length and truncated proteins was expressed from one UAG-containing mRNA. Similarly, Liu and coworkers prepared a ribosome with the C-terminal domain of ribosomal protein L11 to decrease RF-1-mediated translation termination; however, the complete reassignment of the amber codon was not shown [77]. Conversely, Sakamoto's group [78–80] and Wang's group [81, 82] independently reported using a different approach to knock out RF-1 from *E. coli* strains, and both groups demonstrated complete reassignment of the amber UAG codon from translation termination to nonproteinogenic amino acid incorporation *in vivo*. In Wang's approach, the RF-1 knockout strains had a substantially decreased growth rate, most likely because of the failure of translation termination at amber UAG codons in endogenous mRNAs that are essential for the growth of* E. coli*. The combination of this RF-1 knockout approach with the recently reported genome-wide codon replacement method to replace all 314 endogenous UAG codons to UAA stop codons in *E. coli* [83] might solve this problem. Nevertheless, these RF-1 knockout strategies enabled the complete reassignment of the amber codon to a nonproteinogenic amino acid *in vivo*; however, they have only been applied to the amber codon thus far. 

The difficulty of complete codon reassignment *in vivo* clearly comes from the fact that the translation events that compete with the incorporation of nonproteinogenic amino acids are required to maintain the life of the host cells. Conversely, engineering an *in vitro* translation system is not as complicated as for an *in vivo* translation system; even so, the simultaneous complete reassignment of multiple codons to different nonproteinogenic amino acids was not achieved until 2003. For example, Roberts and coworkers used a lysate translation system pretreated with aminoethanol-Sepharose to remove endogenous tRNAs, which compete with the incorporation of a nonproteinogenic amino acid at sense codons. However, a peptide synthesized by using this tRNA-depleted system still consisted of a mixture of proteinogenic and nonproteinogenic amino acids [2]. Other approaches to decrease competitive translation events against nonproteinogenic amino acid incorporation *in vitro* include heat inactivation of RF-1 [84], aptamer-mediated RF-1 inhibition [85], and antibody-mediated RF-1 inhibition [86], yet none of these resulted in complete codon reassignment. Another interesting approach is the pretreatment of a lysate translation system with a phenylalanyl-tRNA synthetase (PheRS) inhibitor, and nearly complete reassignment of the Phe sense codon to naphthylalanine was demonstrated, although its general application to multiple codons has yet to be reported [87]. 

## 5. Genetic Code Reprogramming

**Figure 3:**
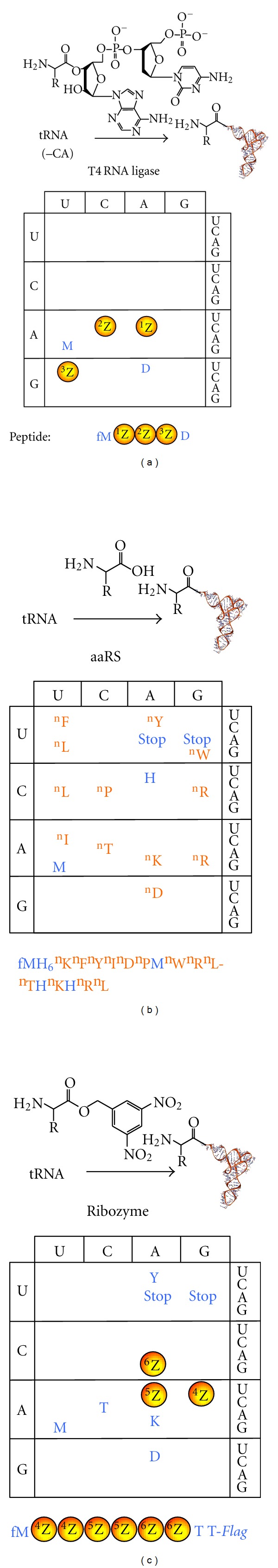
Comparison of the aminoacylation methods used for genetic code reprogramming. (a) Chemoenzymatic acylation method. The dinucleotide carrying the nonproteinogenic amino acid (pdCpA-aa) was enzymatically ligated to tRNA lacking 3′-nucleotides (pCpA). Aminoacyl-tRNAs prepared by the chemoenzymatic acylation method were added to an aaRS-free reconstituted translation system to synthesize a non-standard peptide containing three nonproteinogenic residues (^1^Z, L-propargylglycine; ^2^Z, L-*O*-methylserine; ^3^Z, L-allylglycine). (b) aaRS tRNA-misacylation method. Aminoacyl-tRNAs carrying nonproteinogenic amino acids were enzymatically generated in a reconstituted translation system. A non-standard peptide containing 13 nonproteinogenic residues was synthesized (^*N*^X, an analog of proteinogenic amino acid X). (c) Flexizyme-based acylation method. An activated amino acid was mixed with tRNAs and flexizyme to synthesize aminoacyl-tRNA. Aminoacyl-tRNAs prepared by the flexizyme-based method were added to a reconstituted translation system to synthesize a non-standard peptide containing six nonproteinogenic residues (^4^Z, L-acetyllysine; ^5^Z, L-citrulline; ^6^Z, L-*p*-iodophenylalanine).

The first demonstration of the simultaneous complete reassignment of multiple codons to different nonproteinogenic amino acids (i.e., genetic code reprogramming) was reported by Forster et al. in 2003 (Figure 3(a)) [88]. The key feature of genetic code reprogramming is the use of a reconstituted translation system, instead of crude lysate translation system, to eliminate translation events that compete with the incorporation of no-proteinogenic amino acids. In the report by Forster et al., purified ribosomes, initiation factors, and elongation factors were mixed with fMet-tRNA^ini^, Glu-tRNA^Glu^, and three kinds of chemoenzymatically synthesized nonproteinogenic aa-tRNA which were assigned in the reprogrammed genetic code shown in Figure 3(a), and a non-standard peptide containing three different nonproteinogenic amino acids was synthesized. An interesting feature of their aaRS-free reconstituted translation system is that since orthogonality of the tRNA against aaRS is not required, in principle, tRNA with any body sequence and anticodon can be used to incorporate amino acids [89]. On the other hand, in their genetic code reprogramming system using chemoenzymatic tRNA-acylation, the maximum number of nonproteinogenic amino acids that were simultaneously reassigned in the reprogrammed genetic code was not more than three [88, 90–92], probably because the authors used a technically demanding chemoenzymatic acylation method to prepare the aa-tRNAs [18].

To achieve genetic code reprogramming by a more simple tRNA-acylation method, Szostak's group took advantage of the substrate tolerance of wild-type aaRSs, namely, that “wild-type” aaRSs can charge some proteinogenic amino acid analogs [93]. This feature has been used mainly to incorporate a proteinogenic amino acid analog into proteins *in vivo* (in an auxotrophic strain in many cases) in a residue-specific manner to produce heterogeneous proteins containing proteinogenic and nonproteinogenic amino acids as engineered protein material [94–97]. In the first report of genetic code reprogramming by aaRS-catalyzed acylation, Josephson et al. described a landmark example [93] in which nonproteinogenic amino acids were misacylated onto endogenous tRNAs by wild-type aaRSs in a reconstituted translation system [98] lacking the corresponding proteinogenic amino acids, and 10 different nonproteinogenic amino acids were incorporated simultaneously into a peptide as assigned in the reprogrammed genetic code (Figure 3(b)). Importantly, they also demonstrated that the same non-standard peptide could be displayed on its encoding mRNA via a puromycin linker to show the compatibility of genetic code reprogramming with mRNA display. Later, Szostak's group found that over 90 nonproteinogenic amino acids could be charged onto tRNAs by wild-type and mutant aaRSs and showed that over 50 nonproteinogenic amino acids could be ribosomally incorporated into peptides [99, 100]. They also synthesized a non-standard peptide containing 13 different nonproteinogenic amino acids. In terms of the number of different nonproteinogenic amino acids that can be incorporated simultaneously into ribosomal peptides, this non-standard peptide still holds the record. The advantage of Szostak's reprogramming method is that (1) the misacylated tRNAs can be generated *in situ* under multiple turnover conditions and (2) non-standard peptides can be expressed by merely adding nonproteinogenic amino acids to the translation system from which the corresponding proteinogenic amino acids are withdrawn. On the other hand, the number of available nonproteinogenic amino acids is limited since aaRSs can generally mischarge only those that are structurally similar to proteinogenic amino acids, and it is not possible to introduce multiple analogs of the same amino acid type at the same time (e.g., two proline analogs). Moreover, since proteinogenic amino acids are more acceptable to both aaRS acylation and ribosomal translation, even a tiny amount of proteinogenic amino acid contamination causes the production of undesired product that consists of a mixture of proteinogenic and nonproteinogenic amino acids [93].

As another approach to achieve genetic code reprogramming by a more general tRNA-acylation method, Murakami et al. reported a highly flexible-ribozyme- (flexizyme-) based tRNA-acylation system [101]. In the first demonstration of genetic code reprogramming using flexizymes, three sense codons were reassigned to three nonproteinogenic amino acids charged onto orthogonal tRNAs (Figure 3(c)). According to the reprogrammed genetic code, a 17 mer non-standard peptide possessing six nonproteinogenic amino acids was synthesized in a genetically encoded manner. 

The prototype [102] of the flexizyme used in genetic code reprogramming, which was evolved from a random RNA pool, has expanded its acceptability of tRNAs [103, 104] and amino acids for aminoacylation and turned into highly flexible ribozymes for tRNA aminoacylation [101, 105, 106]. Because the flexizymes recognize their cognate aromatic group, they are able to charge a wide variety of not only L-amino acids with nonproteinogenic side chains [3, 107–111], but also amino acids with an altered backbone such as *N*-alkyl amino acids [112–114], *N*-acyl amino acids [115], D-amino acids [116], *β*-amino acids, oligopeptides [117, 118], and even hydroxy acids [119, 120]. Moreover, as the preparation of aa-tRNAs by flexizymes is simple and straightforward, it should facilitate the parallel preparation of various aa-tRNAs in a high-throughput manner, which enables us to carry out genetic code reprogramming conveniently. In fact, more than 160 kinds of (amino) acids were charged onto tRNAs using the flexizyme system in the past few years. Since flexizymes also recognize conserved 3′-terminal three bases (CCA-3′) of tRNA, they can aminoacylate virtually any tRNA [103, 104]. Recently, Cornish and coworkers took advantage of this tRNA flexibility of flexizymes to aminoacylate base-modified tRNAs with their noncognate amino acids and used the prepared aa-tRNAs to investigate differences in ribosome recognition between the amino acid charged onto cognate and noncognate tRNAs [121]. 

Bearing the advantages and disadvantages of each genetic code reprogramming method in mind, scientists in this research field moved onto the next step to select functional non-standard peptides from genetically encoded non-standard peptide libraries.

## 6. Selection of Functional Nonstandard ****Peptides Generated by Genetic Code ****Reprogramming

The first model study of the *in vitro* display selection of a non-standard peptide prepared with a reconstituted translation system was reported by Forster et al. [122]. Five kinds of biotinylated peptides bearing different lengths of polypeptide spacer were synthesized in a ribosome display format, and biotinylated peptides with a spacer long enough to exit from the ribosome tunnel were selectively pulled down using streptavidin beads. However, in this paper, a diverse non-standard peptide library was not constructed and a novel functional non-standard peptide was not obtained. Very recently, several papers have been published on the selection of functional non-standard peptides by combining genetic code reprogramming with mRNA display selection. In this section, we review the preparation of non-standard libraries and the results of the selection in these reports.

**Figure 4:**
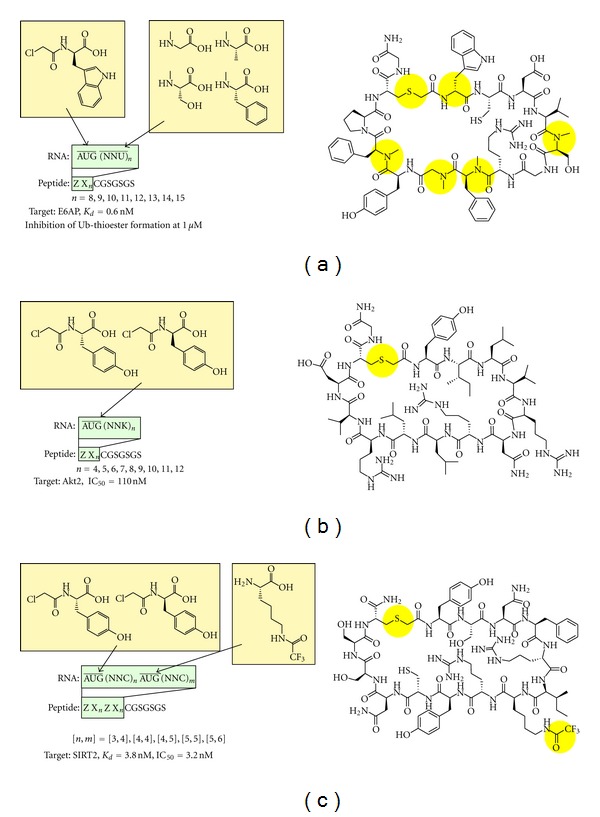
Selection of nonstandard peptides from libraries generated by flexizyme-based genetic code reprogramming. The flexizyme system was used to acylate tRNAs with nonproteinogenic amino acids. The nonstandard peptide libraries were expressed in a reconstituted cell-free translation system in the mRNA display format. (a)Selection of cyclic *N*-methyl-peptides against E6AP. *N*-Chloroacetyl-D-tryptophan was reassigned to AUG, and four *N*-methyl-L-amino acids were independently reassigned to one of the NNU codons. (b)Selection of thioether-cyclized peptides against Akt2. Each enantiomer of *N*-chloroacetyltyrosine was reassigned to the initiation AUG codon. (c)Selection of cyclic peptides containing *N*-6-trifluoroacetyl-L lysine against sirtuin 2 (SIRT2). Each enantiomer of *N*-chloroacetyl-tyrosine was reassigned to the initiation AUG codon and *N*-6-trifluoroacetyl-L-lysine (^CF3^K) was reassigned to the elongation AUG codon.

In 2011, Suga's group published an important study in which highly diverse non-standard peptide libraries containing multiple different nonproteinogenic amino acids were constructed in an mRNA display format, and a novel non-standard peptide was selected from the libraries [5, 123]. Previously, the same group used the flexizyme system to examine the synthesis of various non-standard peptides in a ribosomal translation system [124] and reported the following findings: (1) an *N*-chloroacetyl-amino acid residue and cysteine residue on the same peptide were spontaneously reacted *in situ* using a reconstituted translation system to give a thioether-cyclized peptide without an intermolecular side reaction between the *N*-terminal chloroacetyl group on the peptide and the sulfhydryl group of the other translation component such as a cysteine monomer and DTT [3, 108, 112, 113, 115, 125]; (2) *N*-methyl amino acids with an aromatic side chain or noncharged and nonbulky side chains are efficiently incorporated into peptides by the ribosome, and multiple *N*-methyl amino acids can be incorporated simultaneously into a peptide to give various sequences of thioether-cyclized *N*-methyl-peptides [112]; and (3) the translation initiation apparatus accepts D-amino acids with hydrophobic side chain as relatively good initiators, and pre-*N*-acylation of D-aa-tRNA dramatically increases the efficiency of translation initiation [116]. On the basis of these studies, Yamagishi et al. generated non-standard peptide libraries in which *N*-chloroacetyl-D-tryptophan was reassigned to the AUG start codon and *N*-methyl-phenylalanine, *N*-methyl-serine, *N*-methyl-glycine, and *N*-methyl-alanine were individually reassigned to the UUU, CUU, AUU, and GCU codons, respectively (Figure 4(a)) [5]. The thioether-cyclized *N*-methyl-peptide libraries containing 8–15 random residues were expressed in the mRNA display format by using a translation system reconstituted from a limited set of proteinogenic amino acids and their cognate aaRSs. After reverse transcription, the non-standard peptide libraries were used without any purification for *in vitro *selection against site-specifically biotinylated E6-associated protein (E6AP) together with negative selection using E6AP-free beads. Significantly, unlike the previous report of *in vitro* selection from cyclic peptide library containing an *N*-methyl amino acid with genetic code expansion where the selected peptides did not contain the *N*-methyl amino acid [74], all of the peptides obtained after this *in vitro* selection contained multiple different *N*-methyl amino acids inside the macrocyclized peptide ring. The most abundant cyclic *N*-methyl-peptide among the sequenced clones was chemically synthesized and further characterized. The cyclic *N*-methyl-peptide exhibited high affinity for E6AP (0.6 nM), and despite the fact that the peptide sequence was obtained by affinity selection, it inhibited self-ubiquitination of E6AP *in vitro*. More importantly, removing all of the *N*-methyl groups from the backbone of the cyclic *N*-methyl-peptide dramatically decreased its affinity for E6AP, suggesting that such a cyclic *N*-methyl-peptide is nearly impossible to obtain using conventional approaches, such as the combination of selection from standard peptide libraries and following *N*-methyl modification of the selected peptide. Also, the cyclic *N*-methyl peptide showed 200-times higher affinity than its linear counterpart, which demonstrates that macrocyclization of the *N*-methyl-peptide is important for its binding to E6AP. Moreover, it was shown that both the *N*-methylated backbone and macrocyclization of the cyclic *N*-methyl peptide also contributed to the stability of the cyclic *N*-methyl-peptide against peptidases in human plasma.

The same research group also obtained protein kinase Akt2 inhibitors by mRNA display selection from thioether-cyclized peptide libraries generated by using the flexizyme system (Figure 4(b)) [126]. Peptide libraries containing 4–12 random residues cyclized with either L or D isomers of *N*-chloroacetyl-tyrosine reassigned to the initiation AUG codon were expressed and used for selection against Akt2 immobilized on Ni-NTA beads together with negative selection against Akt2-free beads to remove His-tagged translation components and undesired bead binders. After six rounds of selection, the selected cyclic L- and D-peptides converged to completely distinct sequences, indicating that one configurational difference in the macrocyclized peptides may provide different sequence spaces. In this study, even though enzymatically inactive Akt2 was used for selection, some of the selected peptides showed potent inhibitory activity against active Akt2. On the other hand, despite the fact that the recovery rate after the six rounds of selection between the enriched L- and D-peptide display libraries was at the same level, the most abundant L- and D-peptides showed a different level of inhibitory activity. In addition, the frequency of the selected peptides did not correlate with the inhibitory potency of the selected peptides, indicating the difficulty in identifying the best inhibitor by using only binding-based selection. Even so, the authors successfully obtained potent Akt2 inhibitors by screening chemically synthesized multiple peptides discovered by *in vitro* selection. Interestingly, *in vitro* inhibition assays of the selected L-peptides revealed not only high kinase family specificity but also high Akt isoform specificity, for example, Akt2 (IC_50_ = 110 nM) over Akt1 (IC_50_ ≥ 25,000 nM) and Akt3 (IC_50_ = 4,200 nM) for Pakti-L1 peptide. 

To obtain strong inhibitors by using binding-based *in vitro* selection, Morimoto et al. performed mRNA display selection from cyclic peptide libraries containing *N*-6-trifluoroacetyl-L-lysine (^CF3^K), a nonproteinogenic amino acid inhibitor of its target enzyme (Figure 4(c)) [127]. In this paper, in addition to each enantiomer of *N*-chloroacetyl-tyrosine at the initiation AUG codon, ^CF3^K was simultaneously reassigned to the elongation AUG codon. Thioether-cyclized ^CF3^K-peptide libraries containing 7–11 random residues were prepared and used to select against NAD-dependent deacetylase sirtuin-2 (SIRT2) immobilized on Ni-NTA beads. After six or seven rounds of selection, although various sequences were seen at the randomized positions, the R-I/V-^CF3^K-RY sequence was preferred in the vicinity of the *N*-6-trifluoroacetyl-L-lysine residue. After confirming the inhibition of *in vitro*-selected cyclic ^CF3^K-peptides expressed in the translation system, *in vitro* binding and inhibition assays were performed against two chemically synthesized representative cyclic ^CF3^K-peptides. Importantly, the inhibitory activity of the cyclic ^CF3^K-peptides against SIRT2 correlated well with their binding activity toward SIRT2, and the peptides exhibited high inhibitory activity against SIRT2 (IC_50_ = 3-4 nM). On the other hand, the cyclic ^CF3^K-peptides showed a similar IC_50_ value against SIRT2 to their linear versions (5-6 nM), and the authors showed that the R-I/V-^CF3^K-RY short peptide segment contributed strongly to their SIRT2 inhibitory activity, whereas the constrained macrocyclic structure did not make much contribution. Similarly to the *in vitro*-selected Akt2 inhibitor peptides, *in vitro*-selected SIRT2 inhibitor peptides also showed isoform specificity against SIRT2 (IC_50_ = 3.2 nM) over SIRT1 (IC_50_ = 47 nM) and SIRT3 (IC_50_ = 480 nM). 

**Figure 5:**
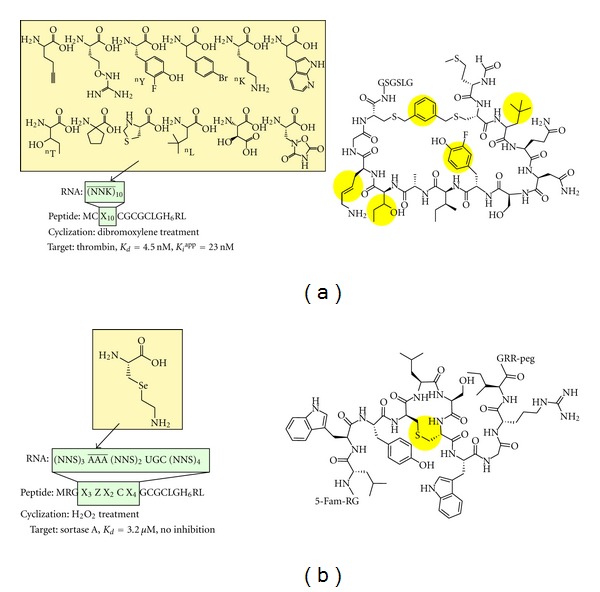
Selection of nonstandard peptides from libraries generated by aaRS-based genetic code reprogramming. Nonproteinogenic amino acids were charged onto tRNA by aaRS misacylation. The non-standard peptide libraries were expressed in a reconstituted cell-free translation system in the mRNA display format. (a) Selection of non-standard peptides from libraries containing 12 different nonproteinogenic amino acids (12 proteinogenic amino acid analogs). The nonproteinogenic amino acids were reassigned to NNK codons. The peptides were cyclized by the addition of 1,3-di(bromomethyl)benzene to react with the sulfhydryl groups on the two cysteine residues of the peptides. The cyclic peptide library, containing 10 random residues, was used for the selection of thrombin inhibitors. (b) Selection of sortase A binding lantipeptides. L-4-selenalysine was reassigned to the AAA codon. The L-4-selenalysine residue was oxidized and converted to dehydroalanine, which was used for cyclization of the peptide. The cyclic peptide library, containing nine random residues, was used for selection.

By taking a different genetic code reprogramming method from Suga's group, Szostak's group demonstrated mRNA display selection from a highly modified non-standard peptide library containing 12 different proteinogenic amino acid analogs (Figure 5(a)) [128]. A peptide library containing 10 random residues between 2 Cys residues was expressed and displayed on encoding mRNAs in the reconstituted translation system with 8 proteinogenic amino acids and 12 nonproteinogenic amino acids. The peptide-mRNA fusion libraries were then cyclized by the addition of 1,3-di(bromomethyl)benzene after oligo-dT purification. After reverse transcription and Ni-NTA purification, the non-standard cyclic peptide library was subjected to selection against biotinylated thrombin protease, and active species were eluted selectively from streptavidin beads using the thrombin inhibitor hirudin. After 10 rounds of selection, several conserved sequences were observed, and importantly, the selected peptides had 50% nonproteinogenic amino acids in sequences that were distinct from the sequences obtained by selection using proteinogenic amino acids. The two most abundant peptides synthesized by the ribosomal translation system showed high affinity (*K*_*d*_ = 4.5 and 20 nM) and inhibitory activity (*K*_*i*_^app^ = 23 and 35 nM) against thrombin, while the parent linear peptides did not. Moreover, the “proteinogenic analogs” of the two peptides did not bind to thrombin (*K*_*d*_ > 500 nM), suggesting that the incorporation of side-chain-modified amino acids may provide a different sequence space from that obtained with proteinogenic amino acids.

By using a similar approach, Hofmann et al. reported mRNA display selection from lanthionine-containing cyclic peptide libraries generated by the ribosomal incorporation of L-4-selenalysine (Figure 5(b)) [129]. 4-Selenalysine was reassigned to the AAA codon by adding 4-selenalysine instead of lysine to a reconstituted translation system, and a library containing nine random residues was generated. After oligo-dT purification, the 4-selenalysine residue was oxidized and converted to dehydroalanine, which was then reacted with the sulfhydryl group on the cysteine residue [130, 131]. After reverse transcription and Ni-NTA purification, the resulting cyclic lantipeptide libraries were used for selection against chemically biotinylated sortase A. After five rounds of selection, several conserved sequences were obtained, and the stereochemistry of the selected lantipeptides was determined through chemical peptide synthesis. While the peptides from one family bound sortase A with an affinity of 3–32 *μ*M, none of the peptides from the other family bound it, suggesting that the sequences in the second family were selected by a different selection pressure from sortase A binding. Moreover, the best sortase A binding peptide did not inhibit its activity.

Taken together, genetic code reprogramming has been successfully combined with *in vitro* display for the selection of non-standard peptide libraries, and it has been shown that most non-standard peptides obtained by this method possess inhibitory activity *in vitro*. Future work will involve demonstrating the activity of the obtained non-standard peptides *in vivo* (not only in cultured cells, but also in an animal model).

## 7. Conclusions and Perspectives

In this paper, we have described the current strategies for the generation of genetically encoded libraries of non-standard peptides and the application of these strategies to the selection of novel functional non-standard peptides. The potential of non-standard peptides has markedly increased following the development of technologies that can generate highly diverse libraries of drug-like non-standard peptides. However, although the selection of several non-standard peptides has been demonstrated, some problems remain to be addressed. One is that there are some limitations to the types of nonproteinogenic amino acids that can be used in a ribosomal translation system. Although the translation system accepts a variety of side-chain-modified amino acids [22, 132], backbone-modified amino acids are less compatible with a ribosomal translation system. For example, Tan et al. showed that D-amino acids (D-alanine, D-phenylalanine) and *β*-amino acids (homo-L-alanine, homo-L-phenylalanine) were not compatible with their reconstituted translation system [133], and Hartman et al. also showed that several *β*-amino acids were not compatible with their reconstituted translation system [100]. We believe that more precise experiments will be required to determine the compatibility of D- and *β*-amino acids with ribosomal translation systems. Following this reevaluation of nonproteinogenic amino acids, further studies on the engineering of translation factors, such as the ribosome [134–136], elongation factor Tu [137, 138], and tRNA [139–141], will be required to overcome these limitations. The second problem is the low cell permeability of peptides. Many potential drug targets are located inside cells, requiring that non-standard peptides have good cell permeability. Since the *N*-alkyl group increases the permeability of a peptide, the incorporation of *N*-alkyl-amino acids into the genetic code might address this problem [112, 113, 142, 143]. Another problem is the fact that the mRNA display method used for selection from highly diverse non-standard peptide libraries is still a laborious and time-consuming technique involving multiple steps. A method to prepare peptide/DNA or peptide/mRNA complex libraries continuously from the corresponding DNA libraries such as CIS display or ribosome display may allow for high-speed library preparation, even though these methods have not been applied to the generation of a highly diverse non-standard peptide library [7, 144]. Nevertheless, it is clear that more improvements of *in vitro* display techniques are required for the development of high-speed non-standard peptide selection. 

Genetic code expansion and reprogramming strategies enable us to generate genetically encoded libraries of non-standard peptides. The value of non-standard peptide libraries will increase as these strategies are developed further. Selection from the genetically encoded non-standard peptide libraries will provide new ways to generate various novel functional peptides that are valuable for biological, therapeutic, and diagnostic applications.
